# Habitat-Associations of Turban Snails on Intertidal and Subtidal Rocky Reefs

**DOI:** 10.1371/journal.pone.0061257

**Published:** 2013-05-10

**Authors:** Amy F. Smoothey

**Affiliations:** Centre for Research on Ecological Impacts of Coastal Cities, Marine Ecology Laboratories A11, School of Biological Sciences, The University of Sydney, Sydney, NSW, Australia; University College Dublin, Ireland

## Abstract

Patchiness of habitat has important influences on distributions and abundances of organisms. Given the increasing threat of loss and alteration of habitats due to pressures associated with humans, there is a need for ecologists to understand species' requirements for habitat and to predict changes to taxa under various future environmental conditions. This study tested hypotheses about the generality of patterns described for one species of marine intertidal turban snail for a different, yet closely-related species in subtidal habitats along the coast of New South Wales, Australia. These two closely-related species live in similar habitats, yet under quite different conditions, which provided an opportunity to investigate how similar types of habitats influence patterns of distribution, abundance and size-structure in intertidal versus subtidal environments. For each species, there were similar associations between biogenically structured habitat and densities. The intertidal species, *Turbo undulates*, were more abundant, with greater proportions of small individuals in habitats formed by the canopy-forming alga, *Hormosira banksii*, the solitary ascidian, *Pyura stolonifera* or the turfing red alga, *Corallina officinalis* compared to simple habitat (bare rock). Similarly, more *Turbo torquatus* were found in biogenically structured subtidal habitat, i.e. canopy-forming algae, *Ecklonia radiata*, mixed algal communities (‘fringe’), or turfing red algae (*Corallina officinalis* and *Amphiroa aniceps*) than where habitat is simple (barrens). Small *T. torquatus* were more abundant in areas of turf and ‘fringe’, while large snails were more abundant in areas of kelp and barrens. These patterns were found at each location sampled (i.e. eight intertidal and two subtidal rocky reefs) and at all times of sampling, across each environment. This study highlighted the consistent influence of biogenically structured habitats on the distribution, abundance and size-structure of intertidal and subtidal turban snails and forms a basis for increasing the understanding of the potential underlying processes causing such patterns.

## Introduction

Patchiness of habitat has important influences on distributions and abundances of organisms that live in a mosaic with different habitats of varying structure and composition [1], [2], [3], [4]. Knowledge of how organisms are distributed in relation to spatial heterogeneity of their environment is fundamental to ecology and has been of concern for many decades (see references in [3]). Despite there being numerous studies done to investigate influences of environmental heterogeneity on a single species in different types of habitat, there have been relatively few comparative tests of such research in different types of habitats for similar species, or among different species across similar types of habitats (e.g. [5], [6], [7]). There is even fewer such comparisons across different environments [8], [9], [10], [11], [12]. Consequently, ecology has been criticised for its lack of progress because of the lack of general ecological models that researchers need to be able to make accurate predictions under changing environmental conditions [13], [14], [15], [16]. In the absence of general predictive models, there cannot be full understanding about ecological requirements of organisms, nor the types of habitats with which they are associated [12], [14], [15], [16], [17], [18], [19], [20].

Intertidal and subtidal rocky reefs are useful habitats for testing hypotheses about the generality of ecological patterns and processes [17], [18], [21]. Despite their apparent complexity and the differences associated with one being partially terrestrial and the other fully aquatic, they have many parallels. For example, each contains a diverse array of organisms and habitats that encompass a large array of environmental conditions which vary over small spatial scales [22], [23], [24], [25]. Thus, each environment is characterized by interspersed patches of varying structure and composition, often only centimetres apart. This may result from physical features, such as pits or crevices on rocky substrata (e.g. [26], [27]), or biogenic structures, such as, algae (e.g. [28], [29]), ascidians (e.g. [30], [31]) and mussels (e.g. [32], [33]). From an animal's perspective, ‘unsuitable’ habitat may be interspersed among ‘suitable’ habitat, thus potentially restricting their distributions. Moreover, anthropogenic disturbances are considered a major threat to marine intertidal and subtidal assemblages [34], [35], [36]. Therefore, mitigating detrimental effects on species, for example due to loss and fragmentation of habitat and, ultimately, to conserve individuals or populations, requires detailed knowledge of the species' habitat-requirements.

At mid to low tidal levels on intertidal rocky shores near Sydney, Australia, *Turbo undulatus*, a relatively common and widespread turban snail, appeared to occur in larger numbers in biogenically structured habitats formed by canopy-forming algae, *Hormosira banksii* (Turner) Decaisne (hereafter *Hormosira*), solitary ascidians, *Pyura stolonifera* Heller (hereafter *Pyura*) or turfing red algae, *Corallina officinalis* Linnaeus (hereafter *Corallina*) than in unstructured habitat – i.e. areas of exposed/open rocky substratum with few crevices, overhangs, pools of water or other biogenic structures created by other macro-algae or barnacles. The first aim of this study was to test the hypothesis that *T. undulatus* were significantly more abundant in these structured habitats than in unstructured habitat. Because heterogeneity of habitat can also influence sizes of marine individuals such as sea urchins (e.g. [37]) and other gastropods (e.g. [38]), this study also aimed to test the hypothesis that the size-structure of *T. undulatus* would differ between structured and adjacent unstructured habitat.

The second aim was to test for generality of patterns of habitat-association across environments and between species. Thus, patterns shown for the intertidal snail *T. undulatus*, were used as a general model, to predict patterns of distribution and size-structure of a closely-related species, *T. torquatus* among similar types of habitat on subtidal rocky reefs. It was, therefore, predicted that the relative patterns of distribution and size-structure of *T. torquatus* between structured and adjacent unstructured subtidal habitat would be the same as those shown by *T. undulatus*. Temperate subtidal rocky reefs are characterised by great heterogeneity in habitat-structure, with mosaics of different habitats, such as kelp, foliose algal turfs and encrusting algae or barrens [22], [23], [39], [40]. Intertidal rocky shore habitats are the interface between terrestrial and aquatic environments and are potentially harsher environments than are subtidal reefs [41]. Comparative tests of patterns of habitat-associations between the two environments will provide a basic framework from which to better understand the influences of habitat on the dispersion of the snails and determine whether similar processes operate to create similar habitat-associations, irrespective of their environment. For comparative purposes, in each of these environments there are: (i) species of *Turbo* and (ii) similarities in the types of habitat (i.e. biogenically-structured and unstructured habitat). Here, subtidal biogenically structured habitats were patches containing either the canopy-forming algae, *Ecklonia radiata* (hereafter kelp), a ‘fringe’ habitat (with mixed algal communities) (hereafter ‘fringe’), or turfing red algae (*Corallina officinalis* and *Amphiroa aniceps*) (hereafter turf). Unstructured habitat was barrens defined as areas of open rocky reef covered in crustose coralline algae (>75%). Filamentous and turfing algae were largely absent, covering <10% of the substratum (as defined by [23]). The final aim was to test that the patterns of abundance and size-structure originally found for *T. undulatus* and *T. torquatus* would be consistent both in time and space.

## Methods

*Turbo undulatus* (Solander, 1786; maximal shell-width ∼40 mm), is relatively common in mid to low tidal areas on intertidal rocky shores in New South Wales, Australia. *Turbo torquatus* (Gmelin, 1791; maximal shell-width ∼100 mm) is a large snail commonly found on shallow subtidal rocky reefs of south-east and south-west Australia, except for Victoria and Tasmania, at latitudes above 28° S [42].

**Figure 1:**
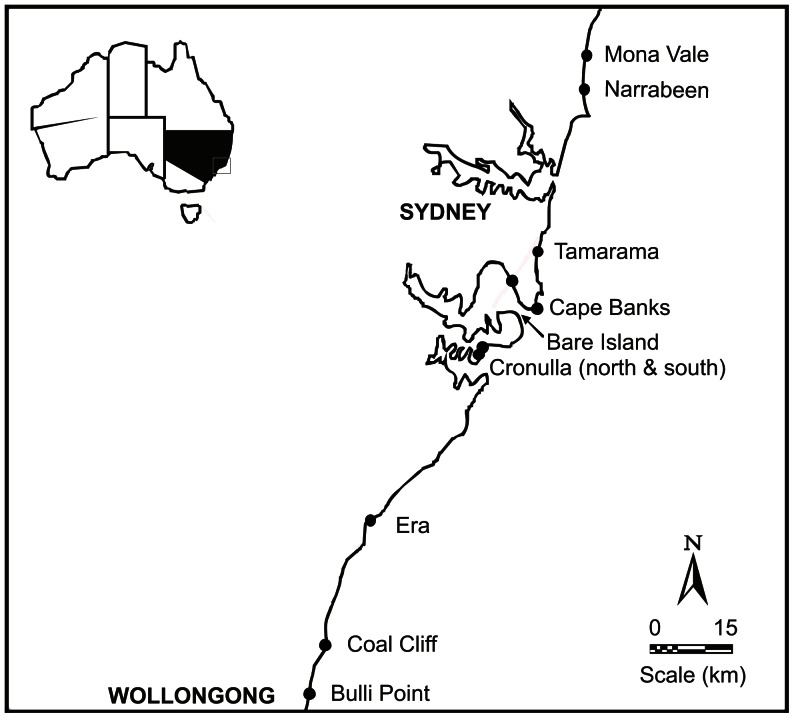
Map of locations studied.

Eight intertidal rocky shores (Figure 1; NSW Fisheries research permit F96/146-6.0) were selected where *T. undulatus* were abundant (>10 per 0.25 m^2^) and the distribution of biogenically structured habitats was patchy, i.e. there were areas of homogeneous structured habitats interspersed with unstructured habitats. All locations were on the open-coast with medium to heavy exposure to waves.

Subtidal areas were relatively horizontal, sandstone rocky reefs at two locations: Cape Banks Scientific Marine Research Area (hereafter called Cape Banks) and Bare Island (Figure 1). All sites at Cape Banks were heavily exposed to waves from prevailing southerly swells, but sites at Bare Island were generally more protected.

To test hypotheses about abundances and sizes of *T. undulatus* in intertidal habitats, snails were counted and measured in haphazardly-thrown quadrats in areas of structured or adjacent unstructured habitat between 2004 and 2006. Maximum shell-widths were measured to the nearest 0.1 mm using vernier callipers. For the purpose of this study, small individuals were defined to be <14 mm shell-width; large snails were ≥14 mm (derived from clear cohorts in the size-frequency histograms of populations of snails across all sampled intertidal habitats). To examine differences in the proportion of small and large *T. undulatus* within, and between structured and adjacent unstructured habitat, data were analysed with Chi-squared tests. Each intertidal habitat was studied initially at one of three locations: Coal Cliff (*Hormosira*), Narrabeen (*Pyura*) and Mona Vale (*Corallina*) (Figure 1) because not all habitats were present at every location (Table 1). To test the generality of the patterns, two additional rocky shores for each type of habitat were sampled (except for *Corallina* which had one). The size and number of quadrats varied depending on the natural variation in population distributions (Table 1). Two independent sites (>10 m^2^, separated by approximately 10 s of metres), each with structured and unstructured habitat, were sampled at most locations (see Table 1 for exceptions), each at similar mid-low tidal heights and levels of wave-exposure.

**Table 1:** Number of quadrats and site(s) sampled in the Sydney Region, NSW, Australia on each intertidal rocky-shore from North to South.+

Study locations	Quadrats	No. of sites	*Hormosira*	*Pyura*	*Corallina*
Mona Vale	6, 50×50 cm	2			✓
Narrabeen	8, 20×20 cm	2		✓	
Tamarama	8, 20×20 cm	2		✓	
Cronulla - north	8, 20×20 cm	2		✓	
Cronulla - south	8, 50×50 cm	1	✓		
Era	6, 50×50 cm	2			✓
Coal Cliff	8, 50×50 cm	2	✓		
Bulli	8, 50×50 cm	2	✓		

+ Mona Vale (33°40′33.46″S, 151°18′23.51″E), North Narrabeen (33°42′23.44″S, 151°17′18.1″E), Tamarama (33°53′52.8″S, 151°16′4.8″E), Cronulla – north and south (34°3′26.78″S, 151°9′7.88″E), Era (151°04′E, 34°09′S), Coal Cliff (34°14′0″S, 150°58′0″E) and Bulli Point (34°19′59.23″S, 150°55′7.14″E; see Figure 1). All locations are on the open-coast with medium to heavy exposure to waves.

✓ indicates the habitat sampled.

On subtidal reefs, *Turbo torquatus* were counted and measured, initially at Cape Banks, in seven haphazardly-placed 5×1 m transects in areas of kelp, ‘fringe’, turf and barrens. This size of sampling unit has been found to give the greatest precision of estimates of densities for *T. torquatus*, in these habitats [43], [44]. Maximum shell-widths were measured to the nearest 0.1 mm. For purposes of this study, small individuals were defined to be <48 mm shell-width; large snails were ≥48 mm. Sizes were derived from clear cohorts in size-frequency histograms of populations of snails across all sampled subtidal habitats. To test the hypothesis that the proportion of small and large *T. torquatus* would differ within, and between structured and adjacent unstructured habitat, data were analysed with Chi-squared tests. Due to the spatial configuration of habitats (i.e. areas of barrens were generally not adjacent to each type of structured habitat in sufficient size), it was not possible for structured and unstructured habitats to be sampled in a site within a location as in the intertidal system. Instead, three independent, haphazardly-chosen sites of each habitat, separated by 10 s of metres, at similar depths and levels of wave-exposure were sampled. To test the hypothesis about the patterns of distribution of *T. torquatus* on subtidal rocky reefs at Cape Banks, data were analysed with a two-factor ANOVA, where habitat was a fixed factor with four levels and site was a random factor with three levels and nested in habitat (*n* = 7). On both times of sampling at Cape Banks (October 2004 & September 2005), variances were heterogeneous and, where possible, stabilized using a fourth root transformation. If variances could not be stabilized, given the relatively robust nature of ANOVA to heterogeneous variances for sampling designs, similar to one used here [45], violation of homogeneity of variances was not considered to be a problem. Nevertheless, results were interpreted with caution, due to the increased probability of Type I error.

To examine the model that the patterns found at Cape Banks are general (in time and space), the same habitat-types were re-sampled 11 months later when Bare Island was also sampled (September 2005). Due to the limited amount of habitat at Bare Island, only two sites of each habitat were sampled and turf was not sampled. Therefore, one site of each type of habitat was removed randomly from Cape Banks, to be comparable with Bare Island and avoid using an unbalanced design. To test the hypothesis that patterns found at Cape Banks would be the same at Bare Island, data were analysed with a three-factor ANOVA, where location and site were random, with two levels each and habitat was fixed with three levels.

## Results

**Figure 2:**
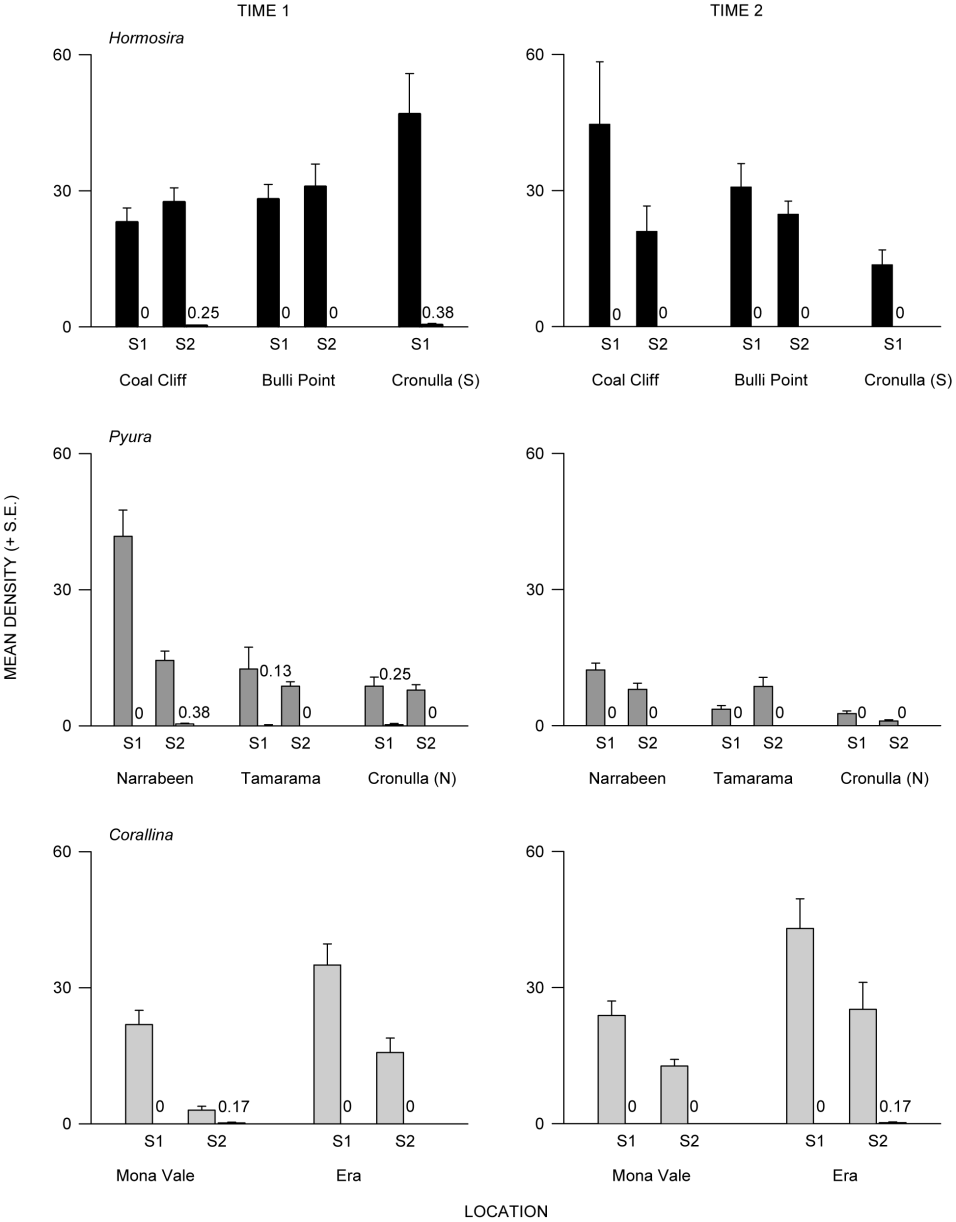
Mean density of *Turbo undulatus* on intertidal rocky reefs. Mean density (+ S.E.; *n* = 8) of *T. undulatus* in areas of (a) *Hormosira* (black bars; quadrat 0.25 m^2^) and non-*Hormosira* (white bars), (b) *Pyura* (dark grey bars; quadrat 0.04 m^2^) and non-*Pyura* (white bars) and (c) *Corallina* (*n* = 6; quadrat 0.25 m^2^, grey bars) and non-*Corallina* (white bars), at each location and at each time of sampling. In areas of unstructured habitat (e.g. non-*Hormosira*), mean density of individuals are presented above the columns, where needed, due to the small number of individuals.

There were more *T. undulatus* in structured than in adjacent unstructured intertidal habitats (Figure 2). In all locations and at all times, only nine individuals were recorded in unstructured habitat, with a maximal density of three snails in one quadrat (0.25 m^2^). In structured habitats, in contrast, *T. undulatus* reached a maximal density of 121 per 0.25 m^2^ (Figure 2). Given such striking differences in densities, no formal analysis was done.

Of the nine snails in unstructured habitat, six were large. In structured habitats, 61, 13 and 82% of the individuals were small in *Hormosira*, *Pyura* and *Corallina*, respectively (Table 2a, b, c). Thus, on average, there were more small *T. undualtus* (<14 mm shell-width) than large snails in *Hormosira* and *Corallina* (Table 2a, c), whereas in *Pyura*, there were significantly more large than small snails (Table 2b). No formal analyses compared size-frequency distributions between structured and unstructured habitat because of the small number of snails in the latter. Nevertheless, small snails appeared to be largely restricted to structured habitats, although large snails were found in all habitats.

**Table 2:** Analyses of the proportion of small (and thus, large) *T. undulatus* in intertidal, structured habitats at each location and time of sampling.+

		TIME 1				TIME 2			
		No. sampled	Small	?^2^	*P*	No. sampled	Small	?^2^	*P*
(a)	*Hormosira*								
	Coal Cliff	404	0.33	45.78	***	525	0.70	81.62	***
	Bulli Point	410	0.65	38.72	***	394	0.90	250.24	***
	Cronulla - south	375	0.42	8.66	**	169	0.68	22.02	***
(b)	*Pyura*								
	Narrabeen		-			162	0.04	138.89	***
	Tamarama		-			98	0.16	44.40	***
	Cronulla – north	133	0.17	56.91	***	72	0.21	24.50	***
(c)	*Corallina*								
	Mona vale	174	0.79	59.79	***	257	0.88	151.01	***
	Era	304	0.81	118.75	***	557	0.81	211.22	***

+ Due to the limited number of snails in unstructured habitat, they were not analysed.

** *P*<0.01,

*** *P*<0.001 and – denotes no data available.

**Figure 3:**
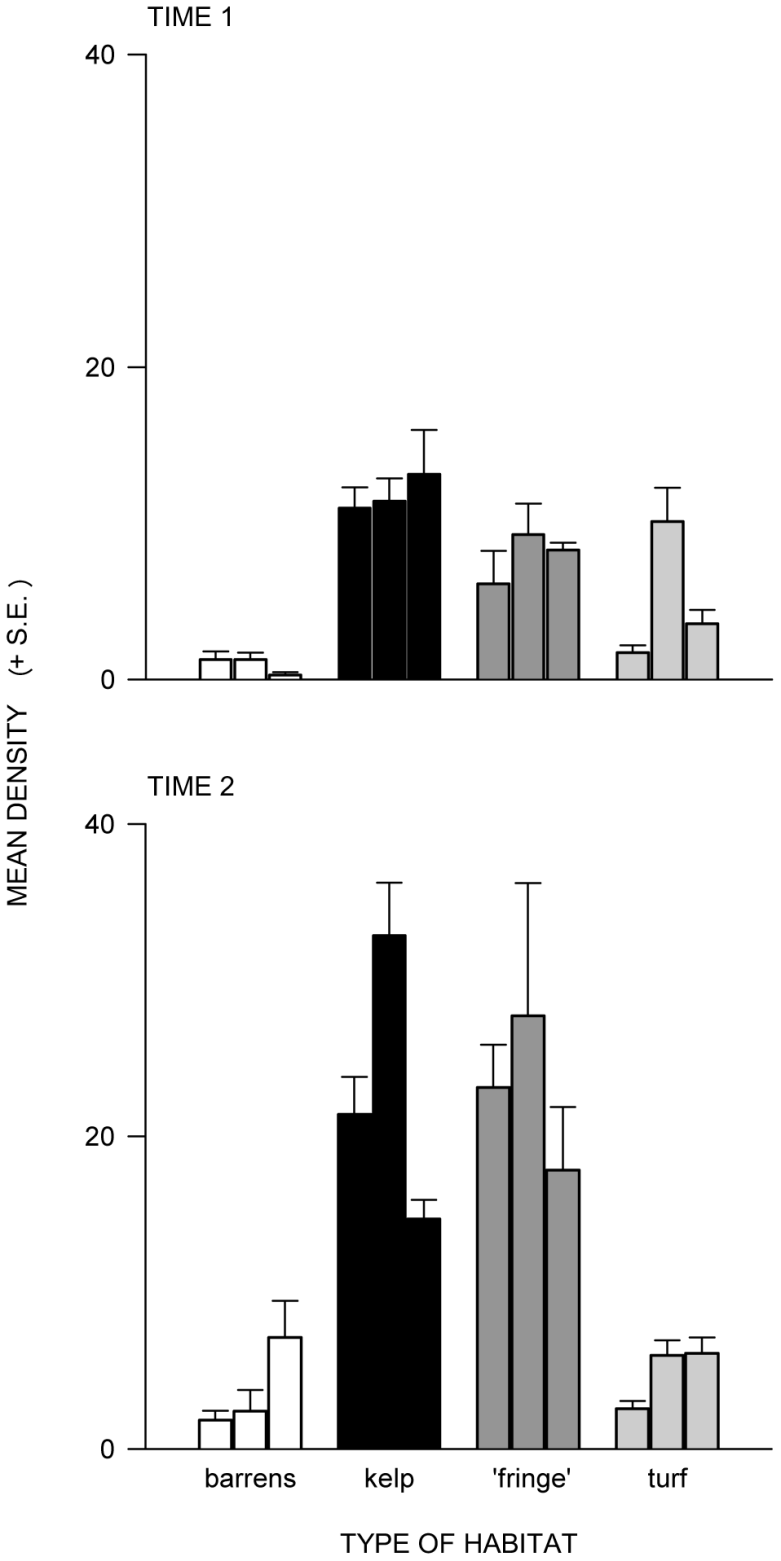
Mean density of *Turbo torquatus* on subtidal rocky reefs. Mean density (+ S.E.; *n* = 7) of *T. torquatus* in 5×1 m transects in each of three representative sites of each habitat at Cape Banks at (a) time 1 and (b) time 2.

Overall, there were greater densities of *T. torquatus* in areas of kelp, ‘fringe’ or turf than in barrens (Table 3, Figure 3), despite variability among sites. There were also significantly more small *T. torquatus* (<48 mm shell-width) than large snails in ‘fringe’ or turf (Table 4a), whereas in barrens or kelp, there were significantly more large than small snails (Table 4a). Spatial patterns of abundance and size-structure of *T. torquatus* the second time of sampling at Cape Banks were consistent with the first time, although there was no difference in densities of snails between barren and turf habitats (Tables 3, 4a; Figure 3). In addition, areas of kelp and ‘fringe’, had greater densities of snails during time 2 than time 1. When the spatial generality of the patterns was tested at Bare Island, similar results were found; more in structured than in unstructured habitat (Tables 3b, 4b).

**Table 3:** Analyses of densities of *T. torquatus* among subtidal habitats at (a)+Cape Banks during each time of sampling and (b)++Cape Banks and Bare Island.

			TIME 1		TIME 2	
	Source	df	MS	*F*	MS	*F*
(a)	Cochran's Test		a *C* = 0.23 S		a *C* = 0.30**	
	Habitat = Ha	3	5.91	12.67**	5.30	21.54***
	Site(Ha) = Si(Ha)	8	0.47	2.87*	0.25	1.88 NS
	Residual	72	0.16		0.13	
	SNK		B<T = F = K		B = T<F = K	
(b)	Cochran's Test		*C* = 0.20 NS			
	Lo	1	42.86	6.50*		
	Ha	2	704.62^x^ ^X^	106.93***		
	Si(Lo x Ha)	6	8.64			
	Lo x Ha	2	0.43			
	Residual	72	12.96			
	SNK		B<F<K			

+ Habitat, fixed 4 levels, Site, random, nested in Habitat, 3 levels, *n* = 7 and (b).

++ Cape Banks and Bare Island; Location, random 2 levels, Habitat, fixed, orthogonal, 3 levels, Site, random, nested in (Location x Habitat), *n* = 7. One site of each type of habitat was removed randomly from Cape Banks, for each time of sampling to be comparable with Bare Island.

a Variances were heterogeneous and were stabilised, where possible, using a forth root transformation (X^0.25^). Significant differences in means were compared using Student-Newman-Keuls (SNK) tests. NS denotes not significant,

* *P*<0.05,

** *P*<0.01,

*** *P*<0.001. For SNK comparisons: B, barrens; K, kelp; F, fringe; T, turf.

X Denotes *post-hoc* pooling, *P*>0.25. New *F*-values are given for those tested against the pooled term.

X Tested against Si(Lo x Ha), Lo x Ha and Residual.

**Table 4:** Analyses of the proportion of small (and thus, large) *T. torquatus* in structured and unstructured habitats on subtidal rocky reefs at each location and time of sampling.+

		TIME 1				TIME 2			
		No. sampled	Small	?^2^	*P*	No. sampled	Small	?^2^	*P*
(a)	Cape Banks								
	barrens	20	0.05	16.20	***	80	0.24	22.05	***
	kelp	249	0.36	20.25	***	483	0.22	156.57	***
	‘fringe’	167	0.86	156.50	***	481	0.76	135.19	***
	turf	108	0.99	104.04	***	103	0.95	83.97	***
(b)	Bare Island								
	kelp	139	0.27	28.55	***				
	‘fringe’	84	0.64	6.86	**				

+ ** *P*<0.01 and *** *P*<0.001.

## Discussion

Many studies have shown the importance of structurally-complex biogenic habitats on intertidal or subtidal rocky reefs, for example beds of algae (e.g. [40], [46], [47]), as influences on small-scale patterns of distribution of a diverse array of taxa. Structured, biogenic habitats on intertidal rocky shores consistently influenced patterns of distribution of *T. undulatus*, irrespective of their type and structure (*Hormosira*, *Pyura* or *Corallina*). Specifically, all structured habitats were associated with greater densities of *T. undulatus* than in adjacent unstructured habitat on each of the rocky shores sampled.

Despite major differences between intertidal and subtidal systems, several generalities emerged. The first was the overall consistent difference in density of snails between structured habitats of biogenic origin and unstructured, non-biogenic subtidal habitat. Hence, the spatial *patterns of T. undulatus on intertidal rocky shores were useful, as a general model, to predict patterns of distribution of T. torquatus* among similar habitats on subtidal rocky reefs. Previous studies have also demonstrated the importance of similar types of habitats on the distribution and abundance of *T. undulatus* [48] and *T. torquatus* [44] in New South Wales and elsewhere [49], [50], [51], and for other turbinid gastropods (e.g. *Turbo smaragdus* [52], [53], [54]). For example, Povey and Keough [49] found in Victoria, Australia densities of *T. undulatus* were greater in areas with large covers of *H. banksii* than elsewhere. *T. torquatus* demonstrated similar spatial patchiness in subtidal habitats in Western Australia, being more abundant in areas of ‘flat reef’ (with *E. radiata* and *Sargassum* spp., [55]) than ‘rock face’ (defined as steeply sloping rock surfaces at the base of steep rock faces; [50]). Fowler-Walker and Connell [56], however, found greater abundances of *T. torquatus* in areas devoid of macro-algae on subtidal reefs in eastern and southern Australia. Their choice of sampling unit (i.e. size and number of quadrats; 1 m^2^; *n* = 6) was chosen to sample kelp and may not have been appropriate for turbinid gastropods and their estimates of abundances are likely to be imprecise compared to studies that have used larger sampling units.

Of the few comparative studies to test hypotheses about consistency of patterns of distribution, abundance and composition of individual species or assemblages between different environments, most have, however, shown patterns to differ. For example, Fielding et al. [57] showed that the macro-invertebrates associated with *P. stolonifera* on intertidal and subtidal rocky reefs along the coast of South Africa differed, despite 42 taxa being common to both. This difference was largely attributed to differences in species composition associated with the *P. stolonifera* in the two environments. On intertidal shores, polychaetes were the dominant invertebrates, whereas crustaceans were the dominant subtidal group [57]. Similar to patterns found here, the snail *Bembicium auratum* were in greater densities in areas of biogenic structure; oysters opposed to bare rock on rocky shores and in mangrove forests in NSW [9], [58].

Another striking pattern found between the two types of habitats in intertidal and subtidal environments was in sizes of snails. In general, small *T. undulatus* were found more in intertidal biogenically structured habitats than were large snails; areas of unstructured habitat had very few individuals smaller than 8 mm. This is consistent with the results of Worthington and Fairweather [48], which showed that *T. undulatus* from areas without coralline algae were larger than from areas with algae. Small *T. torquatus* had larger densities in areas of turf and ‘fringe’, while large snails had greater densities in areas of kelp and barrens (see also [44]).

The confidence with which results can be generalised depends on whether the patterns found are consistent in space and time. In some cases where spatial generality of patterns has successfully been tested (e.g. [59], [60], [61]), patterns varied from place to place, but there was also much variability at the smallest spatial scales (e.g. [62]). Nevertheless, small-scale variability can still be consistent at large scales. In this study it was found that patterns of densities and sizes of turban snails between structured and unstructured habitats initially discovered on a few shores, in the Sydney region, were consistent over other intertidal and subtidal location(s). Moreover, by sampling a second time to assess the precision with which these models can be extrapolated, spatial and size-structure patterns in each system were found to be consistent from one time to the next. Differential use of habitats by organisms has been demonstrated over shorter time-scales than those examined in this study, for example due to the state of tide, time of day, or conditions of weather. The present intertidal study was only done during day-time low-tides, although casual observations during high-tide in areas with *Hormosira* suggested no difference in patterns of *T. undulatus* between high and low tide. *T. torquatus* in contrast lives subtidally and, to show if there was any potential influence of environmental variables on the patterns of *T. torquatus*, sampling of each type of habitat was, as far as possible, stratified for state of tide, time of day (morning vs afternoon) and weather-conditions (calm vs rough).

Given that scales of variability of spatial and size-structure patterns can help to identify the scales of processes influencing patterns, the physical and biological processes determining the patterns of difference found in this study are most likely general to intertidal and subtidal habitats at all locations sampled [63]. For example, differences in spatial and size-structure patterns may be explained by differences in rates of growth and longevity (e.g. [64]), differential patterns of recruitment (e.g. [65]), differential rates of mortality (e.g. [66]), or movement in relation to features of the habitats (e.g. [67]) or physical characteristics of the habitats on the spatial distribution of the organisms (e.g. [68]). While these processes may act in isolation of one another, they may also interact to create the observed patterns.

The consistent small-scale variability in patterns of snails found here (e.g. *Hormosira* to non-*Hormosira* cms to ms apart) suggests that the environmental cues that govern these patterns are more likely to alter spatial variation in abundance via behavioural processes, rather than via recruitment and/or mortality [69]. Associations with structured habitats may, therefore, be due to active movement of the snails in relation to intrinsic differences between the structured and unstructured habitat. Features of habitat to which the snails may respond include physical differences, such as greater structural complexity of these habitats relative to surrounding areas (e.g. [70]) and/or biological characteristics, such as food, directly or indirectly (e.g. [71]), or the ‘local environment’ where the habitat is found (e.g. height on the shore, [72]).

Despite generalities being difficult to identify because of large and complex spatial and temporal variability in responses of organisms across a range of scales [73], this study has shown very striking and predicable spatial patterns of density and sizes of each species of snail with respect to the presence of structured habitats. This was achieved through the use of formal hypotheses, rigorous sampling designs and ensuring that information being compared was commensurable (i.e. in each of these environments there are: (i) species of *Turbo* and (ii) similarities in the types of habitat). Theoretically, differences in the methods of sampling could have confounded these comparisons (in most cases, *Turbo undulatus* were sampled in 0.25 m^2^ and *Turbo torquatus* in 1 m^2^; which were chosen to reflect natural variation in population distributions). There was, however, no evidence that methods of sampling caused problems, because patterns were generally similar. To determine whether similar features of habitats influence the snails in similar ways, irrespective of the species (*T. undulatus* or *T. torquatus*) or the environment (intertidal or subtidal), experimental tests of hypotheses about aspects of each species of snail's behavioural responses to some features of habitat, i.e. biological and/or physical characteristics or features associated with the ‘local environment’ where the habitats are found is needed. Therefore, future experimentation using similar comparative approaches, will increase the predictive capacity of ecological research to contribute to issues of conservation and management of these species and their habitats, in the face of increasing anthropogenic pressures.
